# Cortical recovery of swallowing function in wound botulism

**DOI:** 10.1186/1471-2377-8-13

**Published:** 2008-05-07

**Authors:** Inga K Teismann, Olaf Steinstraeter, Tobias Warnecke, Julian Zimmermann, Erich B Ringelstein, Christo Pantev, Rainer Dziewas

**Affiliations:** 1 Institute for Biomagnetism and Biosignalanalysis, University of Muenster, Muenster, Germany; 2 Department of Neurology, University of Muenster, Muenster, Germany

## Abstract

**Background:**

Botulism is a rare disease caused by intoxication leading to muscle weakness and rapidly progressive dysphagia. With adequate therapy signs of recovery can be observed within several days. In the last few years, brain imaging studies carried out in healthy subjects showed activation of the sensorimotor cortex and the insula during volitional swallowing. However, little is known about cortical changes and compensation mechanisms accompanying swallowing pathology.

**Methods:**

In this study, we applied whole-head magnetoencephalography (MEG) in order to study changes in cortical activation in a 27-year-old patient suffering from wound botulism during recovery from dysphagia. An age-matched group of healthy subjects served as control group. A self-paced swallowing paradigm was performed and data were analyzed using synthetic aperture magnetometry (SAM).

**Results:**

The first MEG measurement, carried out when the patient still demonstrated severe dysphagia, revealed strongly decreased activation of the somatosensory cortex but a strong activation of the right insula and marked recruitment of the left posterior parietal cortex (PPC). In the second measurement performed five days later after clinical recovery from dysphagia we found a decreased activation in these two areas and a bilateral cortical activation of the primary and secondary sensorimotor cortex comparable to the results seen in a healthy control group.

**Conclusion:**

These findings indicate parallel development to normalization of swallowing related cortical activation and clinical recovery from dysphagia and highlight the importance of the insula and the PPC for the central coordination of swallowing. The results suggest that MEG examination of swallowing can reflect short-term changes in patients suffering from neurogenic dysphagia.

## Background

Swallowing is a complex function involving 5 cranial nerves and 50 different smooth and skeletal muscles. In the neural processing of swallowing both brainstem and cortical areas are involved. In the last few years an increasing number of studies has examined the cortical representation of human swallowing by means of fMRI, PET, TMS, and MEG [1-3]. In summary, bilateral involvement of the sensorimotor areas as well as insula activation was found. Most of the studies in this field focussed on healthy subjects; little is known about cortical changes and compensation mechanisms in swallowing pathology.

Due to its high temporal and reasonable spatial resolution [4] MEG is a reliable method for the examination of the complex function of swallowing in humans [3,5]. In the present study we applied whole-head MEG to examine changes in cortical swallowing processing in a patient suffering from wound botulism. Botulism is a muscle-paralyzing disease caused by a neurotoxin produced from the anaerobic, spore-forming bacterium Clostridium botulinum [6]. Botulinum neurotoxin is considered to be the most potent lethal substance known. A severe rapidly progressive dysphagia is one of the earliest symptoms of intoxication. It develops due to weakness of the swallowing muscles and often necessitates gastric tube feeding. Apart from dysphagia, botulism also causes double vision, dropping eyelids, slurred speech, and muscle weakness of the whole body. After accurate therapy fast progressive recovery from dysphagia and other symptoms can usually be observed.

A dramatic increment of wound botulism in heroin abusing subjects has been observed in the last ten to fifteen years [7,8]. In 2005 an increasing number of botulism intoxications has been reported in persons with intravenous drug abuse in northwestern Germany [9]. The clinical symptoms observed in about 10 reported cases were all typical for botulism, although the direct verification of the toxin was only successful in one case [10].

In this case report we wanted to study the clinical and neurofunctional changes in swallowing performance and central swallowing processing during remission from botulism intoxication. Botulism toxin affects motor and autonomic nerves by blocking acetylcholine release [11], but not cortical neurons. Thus we expected an increased cortical representation of volitional swallowing as sign for central compensation of the acute peripheral motor impairment.

## Methods

### Subject

**Figure 1:**
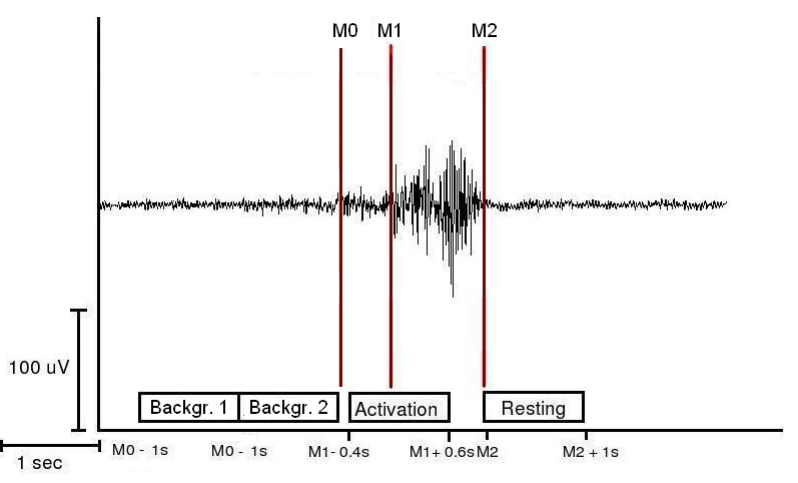
**Definition of the activation and resting stages of swallowing-related muscle activity.** The EMG recording of one representative swallowing act is shown (surface electrodes, recording from the submental muscles). For the SAM-analysis, the starting-point (M1) and the end (M2) of swallowing-related muscle activity were marked. The activation phase and the corresponding resting phase were defined. For estimation of the maximal null distribution and to calculate EMG swallowing power a third marker (M0) was set to distinguish background activity from the beginning of the preparation phase (M_0_) (Methods).

We report the case of a 27-year-old female with a known intravenous heroine abuse for two years. She presented with progressive dysphagia, ptosis, mydriasis, and eye movement disorder on both sides. Computed tomography and magnetic resonance imaging of the head did not show pathological findings. The lumbar puncture revealed a slight dysfunction of the brain-barrier. Direct detection of botulism toxin was negative. On the second day of hospitalization artificial ventilation had to be started due to progression of dysphagia and dyspnoe. Tracheotomy was performed on the third day. Symptomatic treatment conducted on the neurological intensive care unit resulted in fast remission of symptoms within days. Ventilation was stopped two weeks later on the 16^th ^day after admission. However, fiberoptic endoscopic evaluation of swallowing (FEES) [12] then still revealed severe dysphagia due do muscle weakness with pooling and aspiration of saliva. A second FEES examination three days later on the 19^th ^day after admission showed partial recovery of swallowing ability without saliva pooling but extensive residues in the valleculae and sinus piriformes when exposing the patient to food of puree consistency (see Figure 1). The patient was decanulated the same day. At that time she was able to walk for about 10 min without assistance and could speak directly after decanulation. A third FEES examination on the 25^th ^day of hospitalisation showed nearly complete recovery of swallowing ability with the patient being able to eat and drink with little delay in swallowing onset. No residues were seen after the swallow. The patient was discharged from hospital four weeks after admission.

### Control subjects

15 healthy subjects (9 male, age 22 – 34, mean 27.07) served as control group. Control subjects were free of stroke, neuromuscular or neurodegenerative disorders or other conditions potentially being associated with dysphagia. None of them reported swallowing difficulties. When systematically questioned all control subjects denied coughing during eating, slow eating, avoidance of certain food consistencies, fatigable swallowing, or weight loss. One age and gender matched subject also performed a slow swallowing task to imitate the reduced swallowing speed in the patient. By this we wanted to test whether brain changes are driving altered behaviour, or whether the changed behaviour is altering brain activation.

### Dysphagia limit

Before each MEG-recording a dysphagia limit was quantified according to the protocol suggested by Ertekin et al.: Subjects were instructed to swallow increasing bolus sizes of water (1, 3, 5, 10, 15, 20 ml). The maximum bolus size swallowed without piecemeal deglutition was specified [13].

### Swallowing screening test

Before each MEG-recording, a swallowing screening test was performed according to the protocol established by Hughes and Wiles. Subjects drank 150 ml of water from a plastic beaker. They were instructed to drink 'as quickly as comfortably possible'. During the test the subjects were observed from the side, and the number of swallows needed was counted by observing the movements of the thyroid cartilage. A stopwatch was started when the water first touched the bottom lip and stopped when the larynx came to rest for the last time [14].

### Intraoral infusion

To facilitate volitional swallowing during the MEG recording water was infused into the oral cavity via a flexible plastic tube (4.7 mm in diameter) which was attached to a fluid reservoir. The water reservoir bag was positioned about 1 m above the mouth of the subject sitting in the MEG. The tip of the tube was placed in the corner of the mouth between the buccal part of the teeth and the cheek. The tube was gently fixed to the skin with tape. The infusion flow during measurements was about 10 ml/min resulting in a swallowing frequency of about 3 – 5 times per minute.

### MEG recording

During 15 min of MEG recording the subject swallowed self-paced without external cue. One control subject accomplished an additional swallowing task where she was instructed to perform slower swallows with an adjusted slower water infusion. Swallowing acts were recorded and identified by means of electromyographic recording. MEG data were collected using a whole head 275-channel SQUID sensor array (Omega 275, CTF Systems Inc.) installed in a magnetically shielded room. Magnetic fields were recorded with a sampling-rate of 600 Hz. The data were 150 Hz low-pass filtered during acquisition. Recordings were performed while the subject was seated in a comfortable upright position watching a silent movie of her choice. The local ethics committee has approved the protocol of this study, which is part of a research project focusing on neurogenic dysphagia. Informed consent was obtained from all subjects after the nature of the study was explained in accordance to the principles of the Declaration of Helsinki.

### EMG recording

Surface EMG was measured with two pairs of bipolar skin electrodes (Ag-AgCl) placed on the submental muscle groups [15]. The electrodes were connected to a bipolar amplifier (DSQ 2017E EOG/EMG system, CTF Systems Inc., Canada), and the nominal gain was set to 1.

The duration per swallow as well as the RMS of EMG amplitude across the whole swallow interval (M0 – M2) was determined to compare the swallowing muscle activation between the different measurements.

### Data analysis

According to the individual EMG signal the starting point of laryngeal elevation (M_1_) and the end of the task-specific muscle activity (M_2_) were marked for every single swallow during each measurement. The beginning of laryngeal elevation was defined as increase of more than 100% in amplitude or frequency of the EMG signal after an initial increase of EMG activity defining the beginning of the preparation phase. The end of task-specific muscle activity was defined as more than 50% decrease in amplitude or frequency of the EMG signal. To estimate the maximal null distribution (see below) a third marker was set in order to distinguish background activity from the beginning of the preparation phase (M_0_). Only trials with a movement stage longer than 1 s were taken into account. For further analysis, time intervals were defined as follows (see Figure 1):

(1) Movement stage: -0.4 to 0.6 s in reference to M_1_

(2) Resting stage: 0 to 1 s in reference to M_2_

(3) Background stage 1: -2 to -1 s in reference to M_0_

(4) Background stage 2: -1 to 0 s in reference to M_0_

To define the active frequency bands and to examine the temporal sequencing of activation time-frequency plots were calculated using wavelet analysis. Time-frequency plots of parietal and occipital channels were determined and for the control group averaged across subjects. According to SAM analysis, time range from -.4 to .6 in reference to M1 was taken as "execution stage". To demonstrate the desynchronization, this was compared to "predeglutition stage" (-1.4 to – .4).

For SAM analysis MEG data were filtered in five different frequency bands: theta (4–8 Hz), alpha (8–13 Hz), beta (13–30 Hz), low gamma, (30–60 Hz), and high gamma (60–80 Hz). SAM was used to generate 20 × 20 × 14 cm volumetric pseudo-t images [16] from the filtered MEG signals, with 3-mm voxel resolution. A pseudo-t value cancels the common-mode brain activity by subtracting the source power found in a defined control stage from the source power in the active stage. To account for uncorrelated sensor noise, this difference is normalized by the mapped noise power [16,17]. For the analysis of cortical activity during the movement stage (1) the corresponding resting stage (2) served as control condition. The required similarity between the resting stage and the background stages was proven before by a direct comparison of these stages in wavelet und SAM analysis.

Analysis of the control group was performed as previously published [18-21]. Individual MRIs were first transformed into a common anatomical space using SPM2. Then the spatial normalized activation maps were obtained by applying this transformation to the individual SAM volumes. The significance of activated brain regions was investigated by the permutation test method described by Chau and co-workers (2004). The maximal null distribution was estimated by comparing background stage 1 (active) and 2 (control) [21,22].

## Results

The patient tolerated both MEG measurements without any difficulties. No coughing and especially no signs of aspiration were observed. The first measurement was performed on the 20^th ^day after admission, one day after decanulation (see Table 1). FEES still showed signs of severe dysphagia with severe pharyngeal paresis and massive residues but no penetration or aspiration (see above). Dysphagia limit was determined at 5 ml (norm: 20 ml). The swallowing screening-test revealed an abnormally high duration per swallow (4.7 s) as well as an abnormally low volume per swallow (6 ml) and a significantly reduced swallowing capacity (1.27 ml/s). Four days later, when FEES had shown recovery from dysphagia, the second measurement was performed. The dysphagia limit had increased to 15 ml. The swallowing screening-test revealed marked increments in swallowing speed (1 s per swallow), volume per swallow (8 ml) and swallowing capacity (8 ml/s) compared to the test four days earlier. Still the swallowing ability was clearly reduced compared to healthy controls in the terms of the screening-stress-test (see Table 2).

**Table 1:** Time scale

**Day**	**Symptoms**	**Diagnostics and therapy**	**MEG**
1	Dysphagia double vision	Admission on neurologic ICU	
2	Dyspnoe	Intubation	
3		Tracheotomy	
...			
16	Spontaneous breathing	1st FEES examination -> severe dysphagia + aspiration	
...			
19		2nd FEES examination -> persisting dysphagia + no aspiration -> decanulation	
20			1st MEG measurement
...			
25	Further clinical recovery	3rd FEES examination -> mild dysphagia + no aspiration	2nd MEG measurement

Clinical process including symptoms, clinical diagnostics and MEG measurements

**Table 2:** Swallowing screening test

	**1^**st **^measurement**	**2^**nd **^measurement**	**Control group**
**Maximum bolus size (ml)**	5	15	20 +/- 0
**Volume per swallow (ml)**	6	7.89	29.49. +/- 15.25
**Duration per swallow (sec)**	4.72	0.98	1.19 +/- .49
**Swallowing capacity (ml/s)**	1.27	8.06	24.49 +/- 5.21

Results of the swallowing screening tests and dysphagia limit. For the data of the control group mean +/- standard deviation are presented. Swallowing ability of the patient improved dramatically during the 4 days separating the two measurements. Her swallowing ability during the second measurement was still impaired in comparison to the healthy control group.

During the patient's MEG measurements 38 respectively 45 swallows were recorded across the first and second study, respectively. 3 trials had to be rejected in the first measurement due to overlap between the movement stage and the resting stage. In control subjects 69.3 swallows (SD 21.4) were recorded. About 10 % had to be rejected due to stage overlap. The slow swallowing control task resulted in 35 swallows.

The duration per swallow was calculated (1^st ^measurement: 2.22 s mean; 1.05 standard deviation (SD); 2^nd ^measurement: 1.66 s mean; .51 SD). Comparison of the two measurements revealed significantly faster swallowing execution in the 2^nd ^measurement (p < 0.005). Swallowing duration did not differ between the control group (1.94 s mean; .51 SD) and the second patient measurement (p = .462). In the slow swallowing measurement duration per swallow was similar to the first patient measurement (2.26 mean; .67 SD).

RMS of EMG amplitude (M0 – M2) increased between patient measurements (1^st ^measurement: .34 mV mean, .13 SD; 2^nd ^measurement: .42 mV mean; .18 SD, p < 0.005). In control subjects (.7 mV mean, .17 SD) as well as in the slow swallowing task (1.19 mV mean; .26 SD) EMG power was stronger compared to patient's results (p < 0.001).

**Figure 2:**
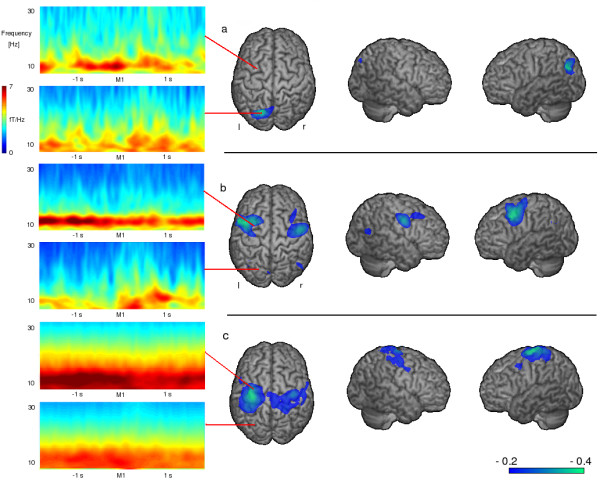
**Time-frequency wavelet plots and event related desynchronizations Wavelet analysis of the parietal and parieto-occipital areas and changes in the beta-frequency-band during swallowing execution.** a) In the first MEG measurement (persisting severe dysphagia) no activation in the somatosensory system can be seen with the chosen threshold. Instead distinct activation of the PPC is found, wavelet analysis shows no systematic activation in either brain region. b) four days later after clinical recovery of swallowing ability distinct activation of the somatosensory cortex with a left hemispheric lateralization was found in wavelet analysis, a distinct beta desynchronisation in the parietal cortex is found in SAM data, while no systematic activation can be seen in the parieto-occipital areas. c) The wavelet results of a healthy control group are similar to those found in the second measurement. Also SAM results reveal distinct beta desynchronization in the parietal cortex. For SAM data the color bar represents the t-value. For wavelet data time 0 on the x-axis corresponds to the individually set markers. Colors represent the level of frequency power, with lower numbers (blue) indicating a decrease in power (ERD) and higher numbers (red) an increase in power (ERS).

**Figure 3:**
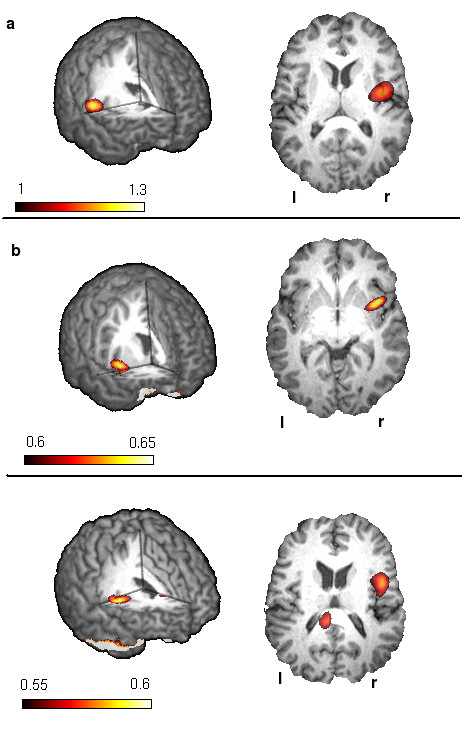
Event related synchronizations Changes in the gamma frequency band during swallowing Please note that the range of the t-value varies between the three figures. a) with persisting dysphagia a strong gamma synchronization of the right insula can be found. b) after clinical recovery of swallowing ability the insula activation has declined. c) In the control group the result is comparable to the results of second patient measurement. The color bar represents the t-value.

SAM analysis of the first patient measurement revealed event related desynchronizations (ERD) in the beta frequency band (13–30 Hz) in the posterior parietal cortex (PPC, Brodman Area 7) with a left hemispheric lateralization (see Figure 2a). Event related synchronizations (ERS) in the right insula were found in the lower (30–60 Hz) and higher (60–80 Hz) gamma frequency band (see Figure 3a). No distinct ERS or ERD could be found in somatosensory areas in any of the 5 calculated frequency bands. Frequency analysis revealed unsystematic activation within the alpha and beta frequency bands in the parietal and parieto-occipital sensors. No distinct event related changes could be observed (see Figure 2a).

The second measurement resulted in ERD of rhythmic brain activity in the beta-frequency-band located bilaterally in the primary and secondary sensorimotor cortex (BAs 4, 3, 1, 2 and 6) with left hemispheric lateralization. The ERD of swallowing related activation was peaking in Brodman area 6 in both hemispheres (see Figure 2b). The right insula showed ERS of the lower and higher gamma band. The amplitude was much weaker compared to the first measurement (pseudo-t value .56 vs. 1.26) (see Figure 3b). The other frequency bands did not show event-related activations in the somatosensory cortex or other brain areas. Wavelet analysis of the parietal areas resulted in activation in the higher alpha and lower beta frequency band and revealed a distinct decrease during swallowing execution. The parieto-occipital areas showed unsystematic activation around the beta frequency band without activation changes during swallowing execution (see Figure 2b).

Control group SAM analysis resulted in ERDs of the beta power showing a distribution comparable to the second patient measurement including the primary sensorimotor cortex (Brodman areas 4 and 1–3) as well as secondary sensorimotor areas (Brodman areas 6 and 40). Again the maximum of the ERD activation was found in Brodman area 6 in both hemispheres. The group pseudo-t was not significantly higher compared to the second patient's measurement (left hemisphere: mean -.52; SD .38 vs. -31; right hemisphere: mean -.48; SD .31 vs. -.24) (see Figure 2c). Again the right insula showed ERS in both the lower and higher gamma band. The group-mean pseudo-t was very close to the second patients measurement (.58 vs .56) and weaker compared to the first patient measurement (.58 mean; .33 SD vs. 1.26) (see Figure 3c). Wavelet results of parietal areas were similar to those of the second patient measurement. No distinct activation changes could be found in the parieto-occipital areas in the control subjects (see Figure 2c).

SAM analysis of the slow swallowing task performed on one control subject resulted in ERD of beta power bilaterally in the primary and sensory sensorimotor cortex with a slight right hemispheric lateralization and weak ERS of the insula region. No activation was found in the PPC of either hemisphere. This result was similar to the control group result.

## Discussion

In this study we observed an increase of insula activation and involvement of the PPC conjoint with decreased activation of the somatosensory system during self-paced swallowing in a patient suffering from severe dysphagia due to botulism intoxication. A second measurement after clinical recovery showed cortical swallowing processing in the primary and secondary sensorimotor system as well as declined insula activation comparable to the results of a group of healthy control subjects.

To distinguish whether the changed swallowing behaviour is altering brain activation or whether cortical changes are driving the altered behaviour a slow swallowing task was performed by one of the control subjects. This imitation of impaired swallowing behaviour resulted in a slight increase of EMG power, while wavelet and SAM results where comparable to those in the normal swallowing task. We therefore conclude that the changed behaviour in deglutition alone cannot explain the observed changes in cortical activation seen in the patient's data.

Surprisingly and contrary to our expectations, very little activation was observed in the sensorimotor system during the first MEG measurement. In contrast, swallowing resulted in strongly left lateralized beta desynchronization of the PPC corresponding to BA 7 and a strong gamma synchronization of the right insula with an amplitude twice as high as in the second measurement and in the control group. We suppose that the increased activation of the insular cortex as well as the PPC activation were part of a compensation mechanism balancing the reduced activation of the somatosensory system.

One possible explanation for the decreased activation of the somatosensory cortex may be that it critically depends on autonomous afferents transferring information about position and cooperation of multiple muscles involved in deglutition, which are affected by botulism intoxication. In contrast to the somatic system, acetylcholine is the major transmitter in both afferents and efferents in the autonomous system. An involvement of primary sensory cortex in the processing of autonomous sensations especially for the oropharyngeal area has already been shown by Penfield and Rasmussen in 1950 [23]. Lack of this autonomous input might lead to a strongly decreased activation of corresponding cortical areas. The effect of botulism toxin on cholinergic autonomous synapses is known to last much longer than the clinically observed symptoms [24]. This might explain the reduced cortical activation during the first measurement with dysphagia having been partially revolved already.

Another explanation for the extremely reduced somatosensory activation may consist in a prolonged down-regulation of the primary motor cortex. Inhibition of the motor cortex due to peripheral disorders has been shown by means of fMRI in muscle fatigue [25]. We may therefore hypothesize that in the patient reported here, botulism related muscle weakness caused an analogous inhibition of primary motor cortex that outlasted the actual time of paralysis.

Interestingly, apart from the reduced activation of the sensorimotor cortex, SAM analyses revealed a predominantly left hemispheric activation of the PPC and an increase in insula gamma synchronization.

The insula has reciprocal connections to many different brain regions including the primary motor, the premotor as well as the primary and secondary sensory cortices [26]. It has been considered to be important in integrating the systems when swallowing is difficult [27]. Mosier and coworkers performed an fMRI study focussing on cortical networks in volitional swallowing [28]. Their results suggest a negative influence of the insula on the somatosensory cortex. On the other hand a parallel loop is proposed consisting of insula, premotor cortex and PPC. These findings are in line with our results showing a strong activation of the insula combined with reduced beta desynchronisation in the somatosensory cortex and a distinct beta desynchronisation of the PPC.

An involvement of the PPC in the processing of swallowing has been shown before by different imaging methods [2,3,29,30]. Principally, the PPC is known to integrate sensory input with motor output [31] and to play an import role in information processing emanating from different sensory modalities [32]. Therefore this sensorimotor integration area is probably crucial in the reception and higher order processing of oropharyngeal sensation, thereby modulating the complex motor activity of deglutition [3]. We assume that in the reported case the PPC additionally to its common tasks also contributes to coordinated muscle activation during swallowing recovery.

The second MEG measurement four days later after clinical and endoscopic recovery from dysphagia showed bilateral sensorimotor beta desynchronisation with a left hemispheric lateralization and a declined gamma synchronization of the right insula during swallowing. Only a small activated region was visible in the PPC during the second measurement. These results correspond to those seen in the control subject of this study as well as in healthy controls of former MEG studies [3,5].

## Conclusion

In conclusion the present results demonstrate a parallel development of clinically observable swallowing function and cortical sensorimotor activation. They provide an additional example of cortical plasticity in response to peripheral dysfunction and highlight in particular the important physiological function of the insula as well as the sensorimotor integration area in human deglutition.

## Competing interests

The authors declare that they have no competing interests.

## Authors' contributions

IT performed analysis and interpretation of data and drafted manuscript and revision. She was funded by the Deutsche Forschungsgemeinschaft. OS has made analysis and interpretation of data and was involved in drafting the manuscript. TW and JZ were responsible for the clinical examination and FEES examination of the patient and for examination of the control group. EBR and CP revised the manuscript critically for important intellectual content. RD made substantial contributions to conception and design, revised the manuscript and has given final approval of the version to be published.

## Pre-publication history

The pre-publication history for this paper can be accessed here:


